# Brain endurance training as a strategy for reducing mental fatigue

**DOI:** 10.3389/fpsyg.2025.1616171

**Published:** 2025-06-18

**Authors:** Nathalie André, Michel Audiffren, Chris Englert

**Affiliations:** ^1^Research Center on Cognition and Learning, University of Poitiers, Poitiers, France; ^2^Department of Sport Psychology, Institute of Sports Sciences, Goethe University Frankfurt, Frankfurt, Germany

**Keywords:** default-mode network, executive functions, fatigability, functional connectivity, mental effort, salience network, sports performance, willpower

## Abstract

Mental fatigue is a psychobiological state triggered by sustained mental effort, affecting subjective parameters, performance, and physiological responses. It impairs sports performance across various disciplines. Individual differences in mental fatiguability and physical fitness may moderate the effects of mental fatigue. Initial evidence suggests that endurance athletes are more resilient to the decrease in the capacity and/or willingness to deploy mental effort induced by mental fatigue, though the results are mixed. Brain Endurance Training (BET) aims to enhance resistance to mental fatigue by combining cognitive and physical training. BET typically uses dual-task designs (simultaneous mental and physical effort), which appear more effective than sequential-task designs. Cognitive tasks involved in brain endurance training often target executive functions, like sustained attention and inhibitory control. While BET consistently improves endurance performance, its effects on subjective mental fatigue are currently less conclusive, which offers intriguing possibilities for future research. Other outcomes, such as perceived exertion and brain oxygenation, suggest BET reduces the cognitive cost of mental and physical effort. BET may also influence brain networks related to attention and self-regulation, particularly the salience network, default mode network (DMN), and frontoparietal network (FPN). Functional connectivity studies hint that BET could lead to beneficial changes in how these networks interact, potentially reducing DMN activity and enhancing control by task-positive networks. Although evidence is still emerging, early findings support BET as a promising intervention to reduce the likelihood of getting mentally fatigued and improve endurance performance in cognitively demanding contexts. Future research should refine BET protocols and explore its underlying neural mechanisms using imaging techniques.

## 1 Introduction

Mental fatigue (MF; Boksem and Tops, 2008) is a psychobiological state caused by prolonged mental exertion and effortful activities (e.g., Marcora et al., 2009). In this context, mental effort refers to the brain mechanism (i.e., a network of brain structures) that allocates resources (e.g., energy, cognitive control) necessary to perform a given task or reach a specific goal (e.g., André et al., 2019; Englert et al., 2021): the longer and the higher the expense in energy and cognitive control, the higher the likelihood of acute MF. Mental effort encompasses the processes involved in concentration, attention, decision-making, and the regulation of emotions while performing the respective task (see also Englert, 2019). MF is thus closely linked to the mobilization of mental effort and is accompanied by changes in subjective parameters (e.g., an increase in the perceived level of exertion; Van Cutsem and Marcora, 2021), performance-related metrics (e.g., increased error rate), as well as physiological correlates (e.g., an elevation in heart rate; Marcora, 2019) and neuronal indicators [e.g., increased activation in the anterior cingulate cortex (ACC); Roelands et al., 2021].

MF can be observed during long cognitive tasks as a function of time on task (e.g., Grier et al., 2003; Pattyn et al., 2008) or in subsequent cognitive or physical tasks in a sequential task protocol (e.g., Giboin and Wolff, 2019; Brown et al., 2020). In the present perspective paper, we assume that acute MF is induced by an accumulation of brain metabolites, byproducts of neuronal activity, in brain regions involved in cognitive control (André et al., 2019; Holroyd, 2024; Pessiglione et al., 2025), which impair brain functioning. Three possible metabolites have been proposed: adenosine (Pageaux et al., 2014; André et al., 2019), beta-amyloid peptides (Holroyd, 2016, 2024) and glutamate (Wiehler et al., 2022; Pessiglione et al., 2025). This accumulation of brain metabolites would lead to a decrease in cognitive control in brain regions involved in executive functions, such as the dorsolateral prefrontal cortex, and in the deployment of effort, such as the ACC. These decreases in cognitive control can be observed with functional brain imaging in the BOLD response of brain structures mediating cognitive control and sustained attention (Salihu et al., 2022) or in the functional connectivity between these structures (Breckel et al., 2013; Esposito et al., 2014; Gui et al., 2015). They can also be observed with electroencephalography with changes in prefrontal theta wave density (Borghini et al., 2014; Tran et al., 2020; Arnau et al., 2021).

As posited by Russell et al. (2021), MF can manifest as an acute condition, triggered by a specific event or exertion; or as a cumulative condition, evolving over the course of a season. The progression of MF is characterized by its impact on performance, which occurs gradually and incrementally. In light of these findings, it is imperative to empower individuals to effectively cope with heightened levels of MF. Empirical evidence has demonstrated the adverse impact of MF on various sports outcomes (see Pageaux and Lepers, 2018 for an overview; see Brown et al., 2020 for a recent meta-analysis), such as endurance performance (e.g., Pageaux and Lepers, 2016), passing accuracy in soccer (e.g., Smith et al., 2016), and decision-making in basketball (e.g., Fortes et al., 2022). However, a recent meta-analysis (Holgado et al., 2020) found that the MF-effect was substantially lower than originally reported, raising the question of which factors might moderate the effects of MF on performance (see also Holgado et al., 2023).

According to Habay et al. (2023), the heterogeneity of results pertaining to MF may be partially attributed to the limited consideration of interindividual differences in existing studies. For instance, Skau et al. (2021) posit that, beyond the level of MF in a given situation (i.e., state level), there are stable interindividual differences in mental fatiguability or MF-susceptibility (i.e., trait level). They suggest that individuals with higher levels of mental fatiguability are more likely to experience MF after tasks requiring mental effort compared to those with lower mental fatiguability. Another potential moderator discussed is the participant’s level of physical fitness (e.g., Jaydari Fard et al., 2019) or sports-specific expertise. In that perspective, two studies reported that athletes with higher aerobic endurance are more resistant to MF effects than individuals with lower aerobic endurance. The first study conducted by Martin et al. (2016) compared the performance of professional and recreational cyclists in a 20-min time trial performed on a cycle ergometer after a modified 30-min incongruent color-word Stroop task (MF condition) or a 10-min control task consisting of fixing a cross located at the center of a screen (control condition). Recreational cyclists produced lower mean power output in the MF condition, while professional cyclists did not. Similarly, Daneshgar-Pironneau et al. (2025) compared the performance of endurance athletes and nonathletes in a time-to-exhaustion handgrip task at 13% of the maximal voluntary contraction performed after a 30-min incongruent Stroop task (MF condition) or a 30-min documentary (control condition). Nonathletes squeezed the dynamometer for a significantly shorter period of time in the MF condition compared to the control condition whereas there were no statistically significant differences between conditions for the endurance athletes. However, other studies (Clark et al., 2019; Van Cutsem et al., 2019) failed to find a training-level effect on MF, possibly due to a lack of statistical power (with only 10 trained athletes and nine badminton players, respectively). In addition, Habay et al.’s (2023) meta-analysis did not show a positive influence of training-level on MF. Moreover, the cross-sectional design of these studies does not allow establishing causality, so future randomized controlled trials are needed to confirm a causal link between endurance training and resistance to MF.

The resistance to MF observed in endurance athletes in the two cross-sectional studies mentioned above is the result of several factors. The gain in aerobic endurance performance following the training of endurance athletes could lead to a reduction of effort costs (e.g., less energy expenditure). However, an increase in the capacity to deploy sustained effort can also explain that endurance athletes are more resistant to MF. Put another way, aerobic endurance can increase the “drive” or willingness to exert effort by decreasing the cost of effort (e.g., perceived effort) and/or by increasing the value of the reward (e.g., effort justification, reinforcement through dopamine releasing) (Schiphof-Godart et al., 2018).

Willpower, a mental skill defined as the capacity to resist temptation through self-control (Baumeister et al., 1998) or to maintain effort despite boredom, fatigue, discomfort, or ego threat (Audiffren et al., 2022), may help explain some of the individual differences observed in MF and endurance performance (for a critical discussion, see Englert, 2025). From this perspective, individuals with greater willpower would be more tolerant to MF and demonstrate superior performance in endurance tasks. Other concepts such as grit, mental toughness, hardiness and resilience, which share similarities with the concept of willpower, have also been viewed as facilitating endurance (e.g., Biggs et al., 2023).

In this perspective paper, we assume that practicing regularly endurance exercise increases willpower by changing the connectivity between brain structures involved in effort regulation, self-control and executive functions such as inhibitory control or planning (Audiffren and André, 2019). A first argument supporting this assumption is that several meta-analyses showed a moderate effect of aerobic exercise on executive functions in older adults (Colcombe and Kramer, 2003; Northey et al., 2018; Sanders et al., 2019; Ye et al., 2024). Similarly, several studies showed that aerobic exercise leads to an increase in gray or white matter volume in brain regions involved in effort regulation and executive functions (e.g., Colcombe et al., 2006; Voss et al., 2013b) and an increase in connectivity between large scale neuronal networks involved in cognition and motor functions (e.g., Voss et al., 2013a; Yan et al., 2024). Training protocols combining aerobic and cognitive exercises in a dual-task design seem to be more promising than aerobic exercise alone to improve attentional capacities, effort deployment and executive functions (Xue et al., 2023). For instance, a study conducted by Anguera et al. (2022) showed that older adults who engaged in a 2-month program integrating simultaneously cognitive and physical training improved on both physical fitness and attentional capacities beyond that of an expectancy matched active placebo control group, with maintenance of improved attention performance evidenced 1 year later. In addition, these authors observed an increase in midline prefrontal theta power density at the end of the intervention only in the group involved in the program combining physical and cognitive stimulation. Prefrontal theta power density is associated with the activity of the ACC, cognitive control and sustained mental effort (Holroyd and Umemoto, 2016). Consequently, “brain endurance training” (BET) that combines endurance exercises and cognitive tasks involving executive functions could serve as a promising intervention to mitigate the onset of MF during or after mentally demanding tasks.

## 2 Brain endurance training

In 2015, Marcora et al. introduced BET, which combines cognitive tasks with physical training to boost resistance to MF and improve endurance performance. Their pioneering study showed that adding cognitive tasks to endurance training extended time to exhaustion more effectively than endurance training alone. Subsequent studies have replicated these findings, confirming the BET’s effectiveness in endurance exercises (Dallaway et al., 2021, 2023; de Lima-Junior et al., 2023; Díaz-García et al., 2024; Staiano et al., 2023).

BET was initially designed to enhance resistance to MF and improve endurance performance. In their seminal study, Marcora et al. (2015) used a dual-task design where participants performed the cognitive task while engaging in vigorous aerobic exercise for 12 weeks. The principal outcome to assess BET effectiveness was a time to exhaustion cycling task at 80% of current VO2max. The comparison of this outcome at the start and end of the intervention in the two groups of participants (i.e., BET vs. endurance training), i.e., the interaction group × time, enables the researcher to estimate the gain in endurance induced by BET. This comparison does not allow the researcher to assess properly the resistance to MF. We will see later the experimental manipulations necessary to assess this second BET effectiveness index.

Over the years, several key parameters of the BET interventions varied across studies: (1) the timing of the cognitive tasks (simultaneously in a dual-task design vs. sequential-task design); (2) the type of cognitive functions that are stimulated during cognitive training; (3) the characteristics of exercise combined with cognitive training; (4) the duration of the program; (5) the outcomes assessed at the start and end of the intervention. These key parameters will be outlined in the following sections.

Regarding the timing of the cognitive tasks during training, four studies used a sequential-task design in which participants performed cognitive tasks in between the physical exercises (Dallaway et al., 2024 – Studies 1–2; Staiano et al., 2023 – Studies 1–2). Three of these studies did not show an increase in the capacity to cope with MF. Given these inconclusive findings, if one of the purposes of BET is to enhance resistance to MF, a dual-task design may be more appropriate than a sequential-task design. During a task, the ACC, involved in the deployment of effort, allocates energy and cognitive control based on the costs and benefits associated with the achievement of the task goal (André et al., 2019; Pessiglione et al., 2025). When the costs outweigh the benefits, participants tend to stop the task or to reduce their resource expenditure (e.g., by decreasing exercise intensity). In this continuous cost–benefit computation made throughout exercise, MF can be viewed as a cost. Training an individual to cope with additional cognitive effort on top of the effort exerted during exercise may enhance their capacity to tolerate higher levels of total effort.

Concerning the type of cognitive functions that are stimulated during cognitive training, Marcora et al. (2015) participants performed a 60-min AX Continuous Performance Task (AX-CPT) while cycling on an ergometer. This task primarily engages executive functions, which are high-level effortful cognitive processes required for tasks that demand attention and concentration when automatic processes are insufficient to cope with the situation (Diamond, 2013). Executive functions are also a prerequisite for self-regulation (Barkley, 2001; Hofmann et al., 2012) and include inhibitory control, cognitive flexibility, updating of working memory, reasoning, problem-solving, as well as planning (Diamond, 2013). Executive function tasks, like the Stroop task, are commonly used to induce MF (Brown et al., 2020) and in BET interventions (see Table 1). Executive functions tasks involved in BET belong to the category of cool executive functions (Zelazo and Carlson, 2012), which are engaged and assessed in emotionally neutral contexts, such as laboratory settings with no motivational incentive. In contrast, hot executive functions that enable behavioral control based on expected rewards or punishments (Zelazo et al., 2010) are engaged in situations which include certain emotional–motivational features. Gambling tasks are commonly used to stimulate or assess hot executive functions in adolescents and adults. The transferability of BET’s gains on resistance to MF is crucial. Transfer distance refers to the similarity between the trained tasks and the tasks used to assess performance improvement at the end of the intervention (i.e., the principal outcome). There are two types of transfer: (a) “near-transfer” effects, where trained and postintervention untrained tasks are similar and (b) “far-transfer” effects, where the tasks differ. The ultimate goal of BET is to promote far-transfer effects because the gain achieved through training must ideally be transferable to competitive sports situations. However, cognitive training with cool executive functions typically leads to near-transfer effects (Audiffren et al., 2022). Combining tasks involving emotional and motivational features and involving hot executive functions with sport-specific skills, such as those used in the Footbonaut (Beavan et al., 2018; Ehmann et al., 2022) or the Skillcourt (Friebe et al., 2023; Erdogan et al., 2024), could help promote far-transfer effects in BET interventions aimed at enhancing resistance to MF in team sport players.

**Table 1:** Overview of studies that have adopted brain endurance training in mental fatigue research.

Study	*N*	NO sessions	NO weeks	Session duration	Intervention content
Marcora et al. (2015)	35	36	12	60	BET: cycling on an ergometer at 65% VO_2_max while performing AX-CPT task CON: Same physical task than BET
Dallaway et al. (2021)	36	26	6	≈ 7	BET: squeezing a handgrip dynamometer once per second at 30% MVC until a pre-determined cumulative force production target while performing cognitive tasks with the non-dominant hand CON: Same physical task than BET
Dallaway et al. (2023)	24	20	5	≈ 7	BET: squeezing a handgrip dynamometer once per second at 30% MVC until a pre-determined cumulative force production target while performing cognitive tasks with the non-dominant hand CON: Same physical task than BET
Dallaway et al. (2024) – Study 1	29	12	4	30	BET: plank, squats, press-ups, wall sit, plank, squats, press-ups, and wall sit + 3-min cognitive tasks after each exercise instead of rest CON: Same physical tasks than BET
Dallaway et al. (2024) – Study 2	29	12	4	30	BET: burpees, plank, jump squats, leg lifts, and press-ups + 3-min cognitive tasks after each exercise instead of rest CON: Same physical tasks than BET
Staiano et al. (2023) – Study 1	28	30	6	NS	BET: 4 cycling sessions and 1 strength and conditioning session + cognitive task after each daily physical training session (30, 45, or 60 min) CON: Same physical tasks than BET
Staiano et al. (2023) – Study 2	25	30	6	NS	BET: 4 cycling sessions and 1 strength and conditioning session + cognitive task after each daily physical training session (30, 45, or 60 min) CON: Same physical tasks than BET
de Lima-Junior et al. (2023)	45	36	12	20-40 min	BET: running at 60% MAV on a treadmill while performing the Stroop task EX: Same physical tasks than BET CON: performing the Stroop task
Díaz-García et al. (2024)	91	30	6	≈ 60 min	BET: 10-min cycling at 60% MHR + 4 series of chest press repetitions to failure at 40% 6RM + 4 series of squat jump repetitions to failure + 15-min cycling at 85% MHR + incongruent Stroop task during the rest periods CON: Same physical tasks than BET

N, Number of participants; NO sessions, Number of training sessions; Session duration, duration of each training session in min; BET, Brain endurance training group; CON, Control group; NS, Not specified; MVC, Maximal voluntary contraction; MAV, Maximal aerobic velocity; MHR, Maximal heart rate.

There are also differences in the type of exercise combined with cognitive training between studies. BET shares similarities with interventions that combine exercise and cognitive training in older adults (e.g., Wollesen et al., 2020; Li et al., 2022; for meta-analyses). However, the goals of these interventions differ. Exercise-cognitive training for older adults aims to improve physical, mental, cognitive or brain health, with the principal outcome typically focusing on physical fitness, quality of life, fear of falling, cognitive performance or gray matter volume, rather than fatigability or endurance performance. Li et al. (2022) coined the term “Exercise Cognitive Combined Training” (ECCT) to designate these broader spectrum interventions that encompass a large range of possibilities: dual-task or sequential-task designs, various physical exercises (e.g., aerobic, resistance, balance, mind–body) and cognitive tasks (e.g., executive functions, prospective memory, information processing speed).

According to the FITT principle, four other parameters of BET interventions need to be discussed: (1) the frequency of the exercise sessions, (2) the intensity of exercises, (3) the duration of each exercise session and (4) the type of exercises. Once again, Table 1 shows that there was a high inter-study variability. Frequency of exercise sessions varied from 3 to 5 sessions per week. The type of exercise varied also a lot across the studies with global aerobic exercises (cycling and running, 5 studies), local isometric exercises (handgrip, 2 studies) and strength exercises (2 studies). The intensity of exercise varied from moderate to vigorous and the duration of exercise sessions from 7 to 60 min. Finally, the duration of BET interventions varies between 12 and 36 sessions, while the total durations range between 4 and 12 weeks. Based on this variety of training protocols, it is imperative to investigate the effects of different BET characteristics on the relevant outcome variables and examine which exercise protocol characteristics lead to larger effect sizes through future meta-analyses.

The typical protocol used to demonstrate the effectivity of BET includes the following elements: (1) a treatment group that practices BET for several weeks; (2) a control group practicing a simple endurance training program for the same duration; (3) a randomization of participants into the two groups; (4) a sequential task protocol performed before the intervention; (5) the same sequential task protocol performed after the intervention. In the interventions presented in Table 1, the sequential-task protocol used to assess MF and endurance performance included two tasks: a long, effortful cognitive task (e.g., 60-min incongruent Stroop task) followed by an endurance task (e.g., time-to-exhaustion task or time trial test). The comparison of endurance performance changes from the start to the end of the intervention in the two groups (i.e., the Time × Group interaction) highlights the endurance gain due to the cognitive training. The assumption is that cognitive training improves resistance to MF while physical training enhances aerobic endurance. To disentangle the psychological and physiological benefits, we propose adding a control session with a low-effort but not boring task (e.g., watching a 60-min documentary), followed by the same endurance task. It is necessary to counterbalance the order of these two sessions within each participant group.

As a final key parameter, the outcomes assessed at the start and end of BET vary between studies. If the main aim of BET is to enhance the resistance to MF in endurance sports, it is crucial to observe the changes in MF symptoms after the intervention while performing an endurance task. The primary outcome for evaluating the effectiveness of BET is thus the endurance performance, measured before and after the intervention. Eight of the nine studies listed in Table 1 showed that the BET group improved more in endurance performance from pre-test to post-test compared to the control group (i.e., endurance training only). However, it was discussed previously that the interaction group × time does not allow to assess the gain in resistance to MF but only the gain in endurance. For assessing the effectiveness of BET on resistance to MF, it is necessary to conduct an analysis of variance testing the interaction group (BET vs. endurance training) × time (pre vs. post intervention) × condition (fatiguing vs. control cognitive task) on the performance of the endurance task. If BET improves resistance to MF more than the endurance program, we can expect that the difference in endurance performance when comparing the fatiguing and the control condition will be larger in the endurance group than in the BET group at the end of the intervention compared to the start.

In addition to the principal outcome, two types of complementary outcomes can be measured to show changes in MF: (1) subjective measures, (2) psychophysiological and neuroimaging measures. Two subjective measures can reflect a decrease in MF: subjective MF assessed with a visual analog scale throughout the sequential-task protocol, and perceived effort during the endurance task. However, self-reports of MF have several limitations (Pessiglione et al., 2025). First, there is a high inter-individual variability in perceiving MF. For instance, Purto et al. (2024) showed that some mining workers underestimate their fatigue, while others do not. Second, participants often confuse MF with other psychological states such as boredom or sleepiness (e.g., Weiler et al., 2025). Third, many studies reported no correlation between objective and subjective measures of MF (e.g., Mehta et al., 2017). In the nine studies in Table 1, only three measured subjective MF (Dallaway et al., 2023; de Lima-Junior et al., 2023; Díaz-García et al., 2024), which yielded inconclusive results. In contrast, perceived effort, measured with the rating of perceived exertion scale (Robertson and Noble, 1997) during endurance tasks, is highly sensitive to the workload (i.e., perceived cost) (Noakes, 2008). This variable was measured in most BET studies, and the results indicate that the BET group reported lower perceived exertion during the endurance task at the post-test compared to the control group (see Table 1; Marcora et al., 2015; de Lima-Junior et al., 2023; Staiano et al., 2023).

Psychophysiological and neuroimaging outcomes help identifying the mechanisms mediating the gains in resistance to MF. Only two studies out of the nine studies in Table 1 used psychophysiological indices of brain changes (Dallaway et al., 2021, 2023). These studies assessed prefrontal cortical hemodynamics using near-infrared spectroscopy (NIRS) and found that, compared to the control group, the BET group showed higher prefrontal oxygenation during the post-test endurance tasks.

## 3 Outlook

The aim of this article was to outline the potential benefits of regular BET on MF resistance. Current research suggests that individuals who undergo BET are less affected by mentally demanding tasks compared to controls. However, research on BET and MF is still in its early stages (see Table 1), and there are no established standards for structuring BET to maximize its benefits. Future research should focus on developing and validating scientific guidelines for conducting BET, distinguishing it from other cognitive-motor training and elucidating the mechanisms that mediate it. Brain imaging could be helpful to examine the brain mechanisms mediating the increase in resistance to MF.

The mechanism of effort that makes decision about resources which need to be deployed during an endurance task is anchored in the ACC and the anterior insula (AI), two key hubs of the salience network (André et al., 2019). When a participant invests effort in a task requiring attention to external stimuli, the right AI helps engaging the central executive network [also called fronto-parietal network (FPN)], which is involved in attention, response inhibition, and working memory, while disengaging the default-mode network (DMN), which is responsible for internally focused thought processes such as self-reflection, daydreaming, mind wandering, recall of personal experiences, and envisioning the future (Menon, 2023). Several brain imaging studies using functional magnetic resonance imaging (fMRI) suggest that changes in the balance between task-positive networks (salience network and FPN) and task-negative network (DMN) are associated with attentional problems and MF.

Brain activation fMRI studies suggest that attentional lapses are linked to increased DMN activation and decreased FPN activation. For instance, greater variability in reaction time was associated with a failure to deactivate the ventromedial prefrontal cortex, a brain region belonging to the DMN, as task difficulty increased (Fassbender et al., 2009). Sustained attention impairments were also tied to increased DMN activation in individuals with traumatic brain injury (Bonnelle et al., 2011). Additionally, MF was associated with heightened DMN activity and reduced FPN activity (Esposito et al., 2014; Gergelyfi et al., 2021). Taking a different approach, Weber et al. (2022) showed that the DMN was more strongly activated during low effort periods compared to high-effort periods, suggesting a gradual regulation of the DMN based on effort costs.

**Figure 1:**
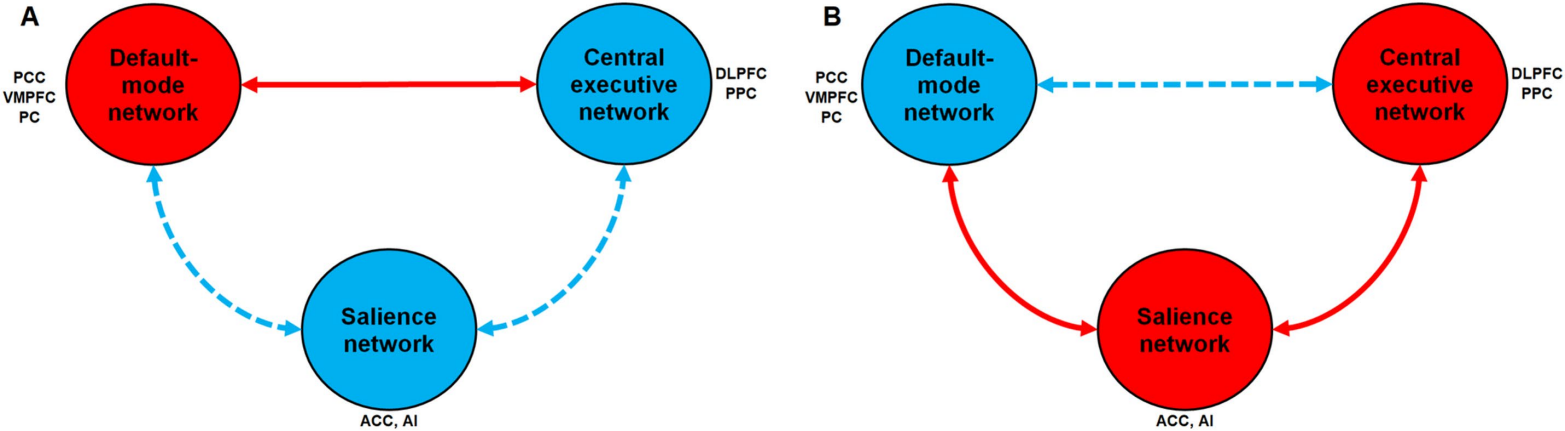
Changes in activation and functional connectivity between the three large-scale neuronal networks involved in cognition according to mental fatigue or brain endurance training. **(A)** Hypothetical changes induced by mental fatigue when comparing the beginning and the end of the sustained attention or endurance task (Time-on-task effect). **(B)** Hypothetical changes induced by brain endurance training on time-on-task effect when comparing before the start and after the end of the intervention. A red circle indicates an increase in activation during the task within this network. A blue circle indicates a decrease in activation within this network. A red solid arrow indicates an increase in functional connectivity (i.e., more synchronization between the two networks). A blue dashed arrow indicates a decrease in functional connectivity (i.e., less synchronization between the two networks). PCC, Posterior cingulate cortex; PC, Precuneus; VMPFC, Ventromedial prefrontal cortex; ACC, Anterior cingulate cortex; AI, Anterior Insula; DLPFC, Dorsolateral prefrontal cortex; PPC, Posterior parietal cortex.

Functional connectivity (FC) fMRI studies suggest that attentional problems due to mental disorders or MF are linked to changes in connectivity between the three large scale neuronal networks (DMN, FPN, and salience network). FC reflects statistical associations between neural activities in different brain regions, indicating synchronized or correlated activity over time. FC does not necessarily imply direct anatomical connections but functional interactions. For instance, Seeburger et al. (2024) found that the activity of the DMN and task positive networks was more anti-correlated during focused attention states (“in-the-zone”) than during suboptimal attentional states (“out-of-the-zone”). During “out-of-the zone” periods, FPN synchronized with the DMN. In contrast, the salience network synchronized more closely with the DMN during “in-the-zone” periods. Earlier, Esterman et al. (2013) showed that during “in-the-zone” periods, extreme peaks in DMN activity were predictive of subsequent errors during a sustained attention task. In contrast, during “out of the zone” periods, reduced activity in dorsal attention network (DAN) and task-relevant sensory regions (parahippocampal place area; PPA) was predictive of subsequent errors. In the same way, Castellanos et al. (2008) showed decreased FC between the ACC (a hub of the salience network), and the precuneus and posterior cingulate cortex (two hubs of the DMN) in people with attention-deficit/hyperactivity disorder (ADHD). More interestingly, Gui et al. (2015) observed an increased FC between the posterior cingulate cortex (a hub of the DMN) and the medial frontal gyrus (a hub of the FPN) over time during a prolonged sustained attention task, reflecting a reduced negative correlation between these regions. Figure 1A summarizes the effect of MF on the three large scale neuronal networks involved in cognition.

The salience network would play a crucial role in the self-regulatory component of endurance performance rather than in its motor component. More precisely, during an endurance exercise, the salience network and more particularly the dorsal ACC would determine the willingness to initiate and maintain goal-directed physical effort (Winterer et al., 2002); e.g., pace of running. In the same way, it is assumed that BET would lead to long-term changes in brain connectivity that would subsequently increase the willingness to keep performing despite increasing sensations of fatigue and pain. BET would stabilize a motivational context necessary for sustaining sequences of actions directed toward specific goals through reinforcement learning (Holroyd and Yeung, 2012). This reinforcement learning would lead to sustainable changes in connectivity within the salience network and between this network and other networks such as the FPN, the DMN and the medial motor network (MMN). Indeed, several studies demonstrated a clear modulation of the activity of the supplementary motor area (SMA), a hub of the MMN, by the dorsal ACC (e.g., Asemi et al., 2015; Diwadkar et al., 2017).

When examining the predicted changes in FC and brain activation induced by a BET intervention, an inverted pattern might be expected (see Figure 1B): a reduced DMN activation during endurance and sustained attention tasks, decreased connectivity between the DMN and the central executive network, and increased connectivity between the salience network and the DMN (i.e., greater control of the anterior insula over DMN nodes). No brain imaging studies have specifically tested this hypothesis yet. However, several studies using fMRI align with this idea. For instance, Kennedy et al. (2022) found increased within-network FC of the salience network and FPN after 10 weeks of mindfulness training. Similarly, Bremer et al. (2022) observed increased FC between nodes of the DMN and the salience network after 31-day of mindfulness meditation training, along with further FC increases between the salience network and key regions of the FPN. Moreover, Leocadi et al. (2024) showed that a 6-week dual-task gait/balance training in Parkinson’s disease patients with postural instability and gait disorders led to increased resting-state FC within the salience network and reduced resting-state FC within the anterior DMN. These results encourage further research in this direction.

The protocol proposed in this article, which includes two interventions (BET vs. endurance training), two time points for assessing endurance (before and after the intervention), and two endurance assessment conditions (after a mentally fatiguing cognitive task vs. after a non-fatiguing control task), is the only one that will clearly highlight the added value of BET in improving resistance to MF. Moreover, the use of brain imaging during the cognitive task preceding the endurance exercise, along with the examination of correlations between, on the one hand, changes in activation and connectivity among major neural networks involved in cognitive control and effort deployment, and on the other hand, gains or losses in endurance performance, will provide a better understanding of the mechanisms underlying the negative effects of MF and the positive effects of BET.

## Data Availability

The original contributions presented in the study are included in the article, further inquiries can be directed to the corresponding author.
